# Effectiveness of anodal transcranial direct current stimulation in patients with chronic low back pain: Design, method and protocol for a randomised controlled trial

**DOI:** 10.1186/1471-2474-12-290

**Published:** 2011-12-28

**Authors:** Kerstin Luedtke, Alison Rushton, Christine Wright, Tim P Juergens, Gerd Mueller, Arne May

**Affiliations:** 1 School of Health and Population Sciences, College of Medical and Dental Sciences, 52 Pritchatts Road, University of Birmingham, Edgbaston B15 2TT, UK; 2 Institute of Systems Neurosciences, University Hospital Eppendorf, Martinistr.52, 20246 Hamburg, Germany; 3 Back Pain Clinic "Am Michel", Ludwig-Erhard-Str.18, 20459 Hamburg, Germany

## Abstract

**Background:**

Electrical stimulation of central nervous system areas with surgically implanted stimulators has been shown to result in pain relief. To avoid the risks and side effects of surgery, transcranial direct current stimulation is an option to electrically stimulate the motor cortex through the skull. Previous research has shown that transcranial direct current stimulation relieves pain in patients with fibromyalgia, chronic neuropathic pain and chronic pelvic pain. Evidence indicates that the method is pain free, safe and inexpensive.

**Methods/Design:**

A randomised controlled trial has been designed to evaluate the effect of transcranial direct current stimulation over the motor cortex for pain reduction in patients with chronic low back pain. It will also investigate whether transcranial direct current stimulation as a prior treatment enhances the symptom reduction achieved by a cognitive-behavioural group intervention. Participants will be randomised to receive a series of 5 days of transcranial direct current stimulation (2 mA, 20 mins) or 20 mins of sham stimulation; followed by a cognitive-behavioural group programme. The primary outcome parameters will measure pain (Visual Analog Scale) and disability (Oswestry Disability Index). Secondary outcome parameters will include the Fear Avoidance Beliefs Questionnaire, the Funktionsfragebogen Hannover (perceived function), Hospital Anxiety Depression Scale, bothersomeness and Health Related Quality of Life (SF 36), as well as Patient-Perceived Satisfactory Improvement. Assessments will take place immediately prior to the first application of transcranial direct current stimulation or sham, after 5 consecutive days of stimulation, immediately after the cognitive-behavioural group programme and at 4 weeks, 12 weeks and 24 weeks follow-up.

**Discussion:**

This trial will help to determine, whether transcranial direct current stimulation is an effective treatment for patients with chronic low back pain and whether it can further enhance the effects of a cognitive behavioural pain management programme. Trial registration: Current Controlled Trials ISRCTN89874874.

## 1. Background

A literature review [1] on the epidemiology and economic burden of non-specific chronic low back pain reported estimates of prevalence ranging from 6% to 11%. In a cross-sectional survey with 9267 respondents, average total back pain costs per patient per year in Germany have been reported as €1322 [2]. Chronic low back pain (CLBP) seems to account for the majority of these expenses, with annual direct costs of > €7000 per patient [1].

Imaging studies have revealed that pain is accompanied by an extensive reorganisation of the brain. Changes in chronic back pain patients are structural [3,4] and functional [5] and reversible if the pain subsides [6-8].

If a chronic pain conditions is regarded as non-specific [9] and does not provide a peripheral tissue target for treatment, the medical treatment approach needs to be directed towards altering these central mechanisms by e.g. prescribing opioids and antidepressants. Surgical interventions include implantation of electrical stimulators in the brain. Deep brain stimulation [10-12], and motor cortex stimulation [13,14] have demonstrated pain reduction.

Transcranial direct current stimulation (tDCS) is a non-invasive alternative that applies weak electrical currents (1-2 mA) through the skull to modulate the activity of neurons in the brain [15].

A current systematic review of the literature (Luedtke et al., Clin. J. Pain, accepted) concluded that anodal tDCS had a pain reducing effect in patients with chronic pain due to spinal cord injury [16,17], fibromyalgia [18,19], chronic pelvic pain [20], multiple sclerosis [21] and various chronic pain conditions [22,23] when applied with an intensity of 1-2 mA over the motor cortex for 20 minutes on a minimum of 2 consecutive days. However, the level of evidence was rated as "low". A risk of bias assessment of the 8 published trials showed that only one trial was of an overall low risk of bias [21]. Four trials met the minimum criteria for the inclusion in the meta-analysis of the results [16,18,21,23]. Although effects for pain were reported as statistically significant across all trials, the pooled effect of -2.29 with a 95% confidence interval of -3.5 to -1.08 only just reached minimal clinically important difference recommendations.

A trial with high methodological quality is needed to determine whether tDCS is effective in the reduction of pain in chronic pain patients.

This trial will evaluate the effect of tDCS on pain and disability of patients with non-specific CLBP and investigate whether tDCS as a prior treatment enhances the symptom reduction achieved by a cognitive-behavioural group intervention.

## 2. Methods/Design

### 2.1 Design

**Figure 1:**
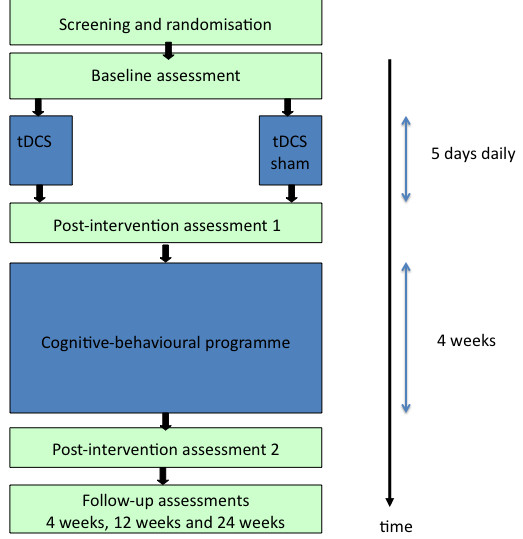
**Trial design**.

Double-blind single-centre randomised controlled trial with two study arms (real and sham stimulation) (Figure 1).

Participants will be allocated to groups (real or sham tDCS) using computer generated randomisation lists, stratified for high (51-100) and low (20-50) average pain intensities over the past 24 hours. Block randomisation (blocks of 20) was chosen to allow for equal numbers in each group at regular time intervals.

Allocation concealment and blinding (patient and therapist providing the intervention) is secured, since the randomisation lists consist of 70 different number codes for each group. Following consent at recruitment, participants will be given the next available code from the randomisation list. The stimulation code will be entered into the tDCS device to initialise the stimulation procedure. According to the entered code the device produces an active or a sham stimulation paradigm. Only the independent researcher (CW) will have access to the list that unblinds the allocation to either tDCS or sham stimulation group.

The stimulation period is followed by a four week cognitive-behavioural group therapy.

### 2.2 Participants

Patients will be recruited and treated at a back pain clinic in North Germany.

Patients will be eligible if they satisfy the following criteria:

• Aged 18 - 65 years

• Categorised as suitable for a pain management programme

• Have non-specific CLBP

◦ with a minimum of 3 months of low back pain without any relevant ongoing pathologies such as acute disc prolapse, acute inflammation, bone fractures, spondylolisthesis or general health restrictions that require medical attention

• Are waiting to attend a cognitive-behavioural group programme at a back pain clinic in North Germany.

• Provide written consent

Potential participants will be excluded if they have any of the following:

• Other chronic pain syndromes

• spinal surgery in the past 6 month

• Neurological disease

• Psychiatric disease

• Does not understand German

• Pregnant or likely to become pregnant during the trial

• Alcohol-, drug-, medication abuse

Medication intake is not listed as an exclusion criterion but type and dosage of medication will be recorded and any change in medication documented.

### 2.3 Interventions

#### tDCS

tDCS is produced by a battery driven small stimulator box and is applied to the skull via sponge electrodes. The equipment is portable and of relatively low cost. Application is easy and safe [24,25]. Across reported trials on tDCS, authors have not observed any serious adverse effects. Side effects have included mild burning or tingling at the site of stimulation, headaches and tiredness [24,26].

All participants will receive 20 mins of anodal tDCS (anode placed over left motor cortex, reference electrode supraorbital on right side) with an intensity of 2 mA, on 5 consecutive days. This dosage has been identified in a systematic review of the current available evidence as the stimulation parameters used in the majority of trials (Luedtke et al., Clin. J. Pain, accepted). Large size (35 cm^2^) sponge electrodes, soaked in a saline solution will be placed over the stimulation sites and held in place by an elastic bandage. The anode (positively charged electrode) will be placed over the left primary motor cortex (M1), while the cathode (negatively charged electrode) will be placed above the right eyebrow. This electrode arrangement has been shown to induce excitability changes in the primary motor cortex [27] and has been used in previous trials on tDCS for the relief of chronic pain [16-23]. The participant will be positioned in supine with legs elevated, during the stimulation.

To accurately determine the site of stimulation, single-pulse magnetic stimuli (TMS) will be applied over the motor cortex on the left side of the skull, until a twitching of the right index finger is observed.

#### Control intervention

An identical procedure will be used for the sham stimulation, but the DC stimulator will not deliver an active stimulation paradigm.

The sham paradigm initially produces a direct current, but switches off automatically after 30 seconds. Participants perceive a tingling sensation identical to that perceived during the real stimulation. This short stimulation does not result in any neurophysiological changes. The method has been shown to be a reliable placebo condition [28]. However, recent studies applying 2 mA stimulations have stated that blinding at this intensity may be less reliable [21]. Therefore blinding will be assessed by asking the participant after the stimulation which mode of stimulation he believes he has received.

#### Cognitive-behavioural group programme

This pain management programme is the standard care for patients at the back pain clinic involved in the study. The effectiveness of interdisciplinary group programmes for the treatment of non-specific CLBP has been demonstrated by a number of publications [29-31]. A maximum of 9 patients per group will receive physically challenging sessions, such as cardiovascular exercises and machine assisted muscle strength training, specific muscle stabilisation exercises for the trunk muscles, as well as information sessions on the neurophysiology of pain, pain coping strategies and relaxation classes. Individual sessions can be added in the case of specific needs, such as acute additional pain that limits the capability to exercise, or biofeedback sessions if a patient finds it impossible to relax in the group sessions. Patients will attend 5 hours of therapy daily (from Monday to Friday) as outpatients.

The programme will be delivered by an interdisciplinary team of orthopaedic consultants, physiotherapists, psychologists and sports therapists who deliver this programme routinely to patients at the clinic.

### 2.4. Outcome measures

#### Primary outcome measures

tDCS and the cognitive-behavioural group programme aim to influence different factors associated with non-specific CLBP. While tDCS is believed to directly influence pain processing within the central nervous system, the cognitive-behavioural programme is targeting disability, beliefs and other psychosocial aspects of the pain experience. Therefore, two primary outcome measures will be used to assess the effect of tDCS as well as of the cognitive-behavioural group programme:

• **Pain**: Visual analogue (VAS 0-100) or numerical rating scale (NRS 0-10) over the past 24 hours. 0 indicates no pain and 100 or 10 indicates the worst imaginable pain.

VAS was used in previous trials on tDCS for the relief of chronic pain [16,18,21]. Minimum clinically important change for visual und numerical pain scales in chronic pain patients has been described as 15 on a 0-100 VAS or 1.5 on a 0-10 NRS [32] or as high as 2.4 in a recent publication [33].

• **Disability: **Oswestry Disability Index [34]. The maximum score is 50 points with a high score indicating a high level of disability.

Validated in German [35]. The cut off point for clinically important change in Oswestry Disability index has been proposed as 10 points [32].

#### Secondary outcome parameters

• **Patient-perceived satisfactory improvement (PPSI): **Participants' perceived global rating of overall change (from baseline) will be recorded on a 5-point Likert-type scale (1 = much worse, 2 = slightly worse, 3 = no change, 4 = slightly better, 5 = much better) [36]. This measurement is a global indicator for change.

• **Bothersomeness: **A second global score that will be used is "bothersomeness". This single question ("how bothersome is your pain today?") tool gives 5 answering options: "not at all," "slightly," "moderately," "very much," and "extremely" [37].

• **General health and health-related quality of life: **SF 36 has been widely used in study populations with chronic pain [38-40]. It has been translated and validated into German [41].

• **Perceived restriction of function: **The Funktionsfragebogen Hannover (FfbH-R) consists of 12 items that ask about the patient's capacity to perform daily life activities. It has been designed to reflect the limitations, a back pain patient may typically experience. The answers are rated in a 3 point scale ("no, can't perform activity", "yes, with difficulties", "yes"). Validity and reliability has been established [42].

• **Fear avoidance beliefs: **Fear avoidance beliefs questionnaire (FABQ) developed by Waddell et al. [43,44] translated into German [44] and evaluated for its psychometric properties [45]. Fear avoidance beliefs has been strongly associated with chronic back pain in the past [46] although its role has recently been questioned [47]. Since patients with a high level of fear avoidance may respond differently to the intervention, it is included in this study as a secondary outcome parameter.

• **Anxiety and Depression: **Hospital anxiety and depression scale (HADS) [48]. Analog to fear avoidance beliefs, depression may hinder the effectiveness of the intervention and needs to be documented for the purpose of this trial.

### 2.5 Sample size

The sample size calculation was based on published minimum clinically relevant change recommendations of 15 mm on a 0-100 mm VAS [32] and 8 points for the ODI [33]. Standard deviations were taken from a previous publication on a comparable study population [49]. With 90% power and α = .01 and anticipated drop-out rates of 10% during the intervention phase and 15% between intervention and final follow-up, the required sample size was calculated as 135.

### 2.6 Data analysis

Data will be digitalised from the paper version (source data) into Microsoft excel spreadsheets by a blinded investigator.

Data analysis will be conducted using SPSS 18 for Apple Macintosh. The primary analysis will use a general linear model to compare between-group effects on the primary outcome measure, at 4 weeks, 12 weeks and 24 weeks follow-up, with baseline values as covariate. Similar analyses will be conducted on appropriate secondary outcomes, or non-parametric tests as appropriate (e.g. on PPSI).

### 2.7 Ethical aspects

Conduct of the study will be in accordance with the Declaration of Helsinki [50]. The project has been approved by the ethics committee of the Ärztekammer Hamburg on 04.01.2010 (responsible body for studies conducted on patients in the region of Hamburg). Ethical approval and was provided by the university ethics committee at the University of Birmingham.

## 3 Discussion

The proposed study presents the first high quality randomised controlled trial on tDCS for the reduction of chronic pain. As identified in a recently published Cochrane review [51] and a systematic review and metaanalysis conducted by our group (Luedtke et al., Clin. J. Pain, accepted), only 8 studies have investigated tDCS for chronic pain reduction [16-23]. None of these was adequately powered to allow valid conclusions on it's effectiveness. Additional risk of bias was introduced by methodological issues, such as invalid randomisation procedures and unclear blinding, leading to a grading of the current level of evidence as "low" (Luedtke et al., Clin. J. Pain, accepted) according to the GRADE system [52]. Computer generated randomisation lists and a tDCS device that produces pre-programmed stimulation paradigms (verum and sham) initialised by 5 digit number codes, will address these issues in the proposed study. The sample size estimation ensures adequate power (90%) to allow valid conclusions regarding the effectiveness of tDCS on the two primary outcome parameters pain intensity (VAS) and disability (ODI).

Chronic low back pain patients have been included in two of the existing trials on various chronic pain conditions [22,23] but no study has exclusively focused on this patient group. With the high prevalence of low back pain and the socioeconomic burden of chronic pain, there is a demand for effective, safe, non-invasive and low cost treatment options for this specific patient group.

The choice of outcome parameters was based on the review of previous publications on comparable study populations/interventions. A range of identified measurement tools was monitored over a 3 month period in the back pain clinic. These included the Short Form McGill Pain Questionnaire [53], the Visual Analog Scale, the Roland Morris Disability Questionnaire [54] and the Oswestry Disability Index [34]. This monitoring phase showed that the Visual Analog Scale and the Oswestry Disability Index had the best responsiveness and the least ceiling effects in the anticipated target population.

However, the study is limited to investigate the short term effects of tDCS and the combination of tDCS and a cognitive-behavioural group programme. Since all patients will receive the group programme following the stimulation period, no long term effects of tDCS alone can be measured. The group programme is currently the standard care for patients attending the back pain clinic and ethical as well as health insurance reasons required that all patients receive the group programme as soon as possible. It was therefore not feasible to include a third group receiving tDCS alone or to postpone the group programme until long term measurements were taken.

## Competing interests

The authors declare that they have no competing interests.

## Authors' contributions

KL participated in the study design and drafted the manuscript. AR participated in the design and helped to draft the manuscript. CW participated in the design, helped to draft the manuscript, and performed the statistical analysis. TPJ participated in the sequence alignment. GM participated in the study coordination. AM conceived of the study, and participated in its design and coordination and helped to draft the manuscript. All authors read and approved the final manuscript.

## Pre-publication history

The pre-publication history for this paper can be accessed here:

http://www.biomedcentral.com/1471-2474/12/290/prepub
